# Knowledge, attitudes, and perspectives on gene therapy for Fragile X syndrome: results from two community and caregiver surveys

**DOI:** 10.3389/fnmol.2026.1912516

**Published:** 2026-07-29

**Authors:** Vivian Chen, Marisa Vomvos, Anna De Sonia, Hilary Rosselot, Reymundo Lozano

**Affiliations:** 1 Department of Genetics and Genomic Sciences, Icahn School of Medicine at Mount Sinai, New York, NY, United States; 2 National Fragile X Foundation, McLean, VA, United States; 3 Department of Pediatrics, Icahn School of Medicine at Mount Sinai, New York, NY, United States

**Keywords:** AAV, caregiver perspectives, Fragile X syndrome, gene therapy, knowledge and attitudes, rare disease, survey

## Abstract

Fragile X syndrome (FXS) is the most common inherited cause of intellectual disability and autism spectrum disorder, resulting from CGG trinucleotide repeat expansion in the FMR1 gene and consequent absence of Fragile X Messenger Ribonucleoprotein (FMRP). Current management remains symptomatic, and adeno-associated virus (AAV)-mediated gene therapy represents a promising treatment and perhaps a curative avenue. However, community perspectives on gene therapy in FXS have not been assessed. FXS is a non-lethal neurodevelopmental condition with a normal life expectancy. Caregivers weighing gene therapy for a child with FXS must balance the potential benefits of FMRP restoration against the uncertainties and risks of an investigational gene therapy in the absence of life-threatening urgency. Whether and to what degree FXS caregivers are willing to consider gene therapy under these circumstances is therefore a meaningful and non-trivial question, and one that has not previously been examined. We report findings from two sequential cross-sectional surveys examining community and caregiver knowledge, attitudes, concerns, and information preferences regarding gene therapy for FXS. The initial survey (Survey One) was an 8-item online survey completed by 351 FXS community members. A follow-up survey (Survey Two) was a 26-item online, REDCap-based instrument completed by 56 parents/caregivers recruited through clinics and the National Fragile X International Conference. Both surveys demonstrated strongly positive attitudes toward gene therapy: 94% of Survey One community members indicated they would consider gene therapy for FXS, while 84% of Survey Two caregivers agreed or strongly agreed they were hopeful about gene therapy success, and an equal 84% indicated they would consider gene therapy if available. The primary concern across both surveys was side effects and risks, followed by effectiveness, timeline, and cost. Improving quality of life was the leading motivator (69%), and intellectual and developmental disability was the symptom caregivers most wanted a future therapy to address (57%). Physicians, genetic counselors, and geneticists were identified as the preferred sources for gene therapy education. These findings reveal that surveyed caregivers express high motivation and broadly positive attitudes toward gene therapy, while highlighting specific educational needs that may inform informed consent processes and clinical trial design for future FXS gene therapy development. These results represent the perspectives of surveyed caregivers and may not generalize to the broader FXS community.

## 1 Introduction

Fragile X syndrome (FXS) is the most common inherited form of intellectual disability and autism spectrum disorder (ASD), affecting approximately 1 in 4,000 males and 1 in 4,000–8,000 females (Hagerman et al., 2017). FXS results from a CGG trinucleotide repeat expansion exceeding 200 repeats in the 5’-untranslated region of the FMR1 gene on Xq27.3, leading to promoter hypermethylation, transcriptional silencing, and loss of Fragile X Messenger Ribonucleoprotein (FMRP) (Milla et al., 2023; Shitik et al., 2020). FMRP is an RNA-binding protein that regulates mRNA translation at the synapse and plays a critical role in synaptic plasticity and neuronal development (Darnell and Klann, 2013). Its absence results in a broad neurodevelopmental phenotype encompassing intellectual and developmental disability, behavioral abnormalities including anxiety, aggression, hyperarousal, and seizures (Protic et al., 2022).

Current FXS management is entirely symptomatic. Behavioral and educational interventions, including occupational therapy, speech-language therapy, applied behavior analysis, and special education, form the foundation of care (Lozano et al., 2016). This is supplemented by pharmacologic management of associated symptoms such as anxiety, attention difficulties, and irritability using agents including selective serotonin reuptake inhibitors, clonidine, stimulants, and antipsychotics, among others (Hagerman et al., 2017; Lozano et al., 2016; Raspa et al., 2023). Despite the widespread use of these interventions, most caregivers report that current symptomatic treatments provide limited functional improvement in the core features of FXS, underscoring the urgent need for therapies that target the underlying etiology (Raspa et al., 2023). Despite decades of research and multiple failed targeted treatment trials, no disease-modifying therapy addressing the underlying genetic etiology of FXS has been approved (Lozano et al., 2016). The unmet need for curative treatment is widely recognized within the FXS community and represents a central priority for affected families, clinicians, and researchers alike.

Gene therapy offers a direct approach to addressing the root cause of FXS by restoring FMRP expression. As defined by the U.S. Food and Drug Administration (FDA), gene therapy encompasses methods of modifying a person’s genes to treat or cure disease (U.S. Food and Drug Administration, 2026). Adeno-associated virus (AAV)-mediated delivery of FMR1 has demonstrated promising results in preclinical models, restoring FMRP expression and improving behavioral outcomes in FXS animal models (Chadman et al., 2023; Hampson et al., 2019; Jiang et al., 2022). AAV-based gene therapy strategies are already in active clinical development for related neurodevelopmental disorders, including Rett syndrome and Angelman syndrome, providing a translational precedent for FXS (Foundation for Angelman Syndrome Therapeutics, 2025; Percy et al., 2024). Given this emerging clinical landscape, AAV-mediated gene therapy for FXS is a realistic near-term prospect, making community preparedness an urgent priority.

A critical and underexplored dimension of this preparedness is caregiver willingness. Unlike conditions that are rapidly progressive or lethal, such as Duchenne muscular dystrophy or certain lysosomal storage disorders, FXS is a non-lethal neurodevelopmental condition with a normal life expectancy (Lozano et al., 2016). This distinction matters: caregivers weighing gene therapy for a child with FXS must balance the potential benefits of FMRP restoration against the uncertainties and risks of an investigational intervention in the absence of life-threatening urgency. Whether and to what degree FXS caregivers are willing to consider gene therapy under these circumstances is therefore a meaningful and non-trivial question, and one that has not previously been investigated (Eley et al., 2025).

Understanding stakeholder perspectives is an essential component of responsible gene therapy development more broadly. The FDA has emphasized that community input informs its evaluation of gene therapies by clarifying how safe, effective, and acceptable a given treatment is likely to be within its intended disease community (Food Drug Administration, 2026). Research across other rare disease communities bears this out: a multinational survey of the Gaucher disease community identified significant gaps in patient knowledge about gene therapy and underscored the need for accessible educational resources (Collin-Histed et al., 2023). Qualitative research in the Duchenne muscular dystrophy community revealed that families placed high value on improving skeletal muscle, cardiac, and pulmonary function, and held nuanced views about the optimal timing of gene therapy initiation (Landrum Peay et al., 2019). A similar study in Friedreich ataxia documented specific community concerns about trial participation and long-term safety (Trantham et al., 2023). Collectively, these studies demonstrate that assessing community perspectives early in the development process yields actionable information about priorities, concerns, and readiness that cannot be inferred from clinical data alone. A concurrent preliminary study has since described caregiver perspectives mostly in caregiver of individuals with FXS from the U.K (Eley et al., 2025); the present surveys extend this work with a larger combined sample from caregivers and the community from the US.

To address this gap, we conducted two sequential cross-sectional surveys examining the knowledge, attitudes, concerns, and information preferences of FXS community members and caregivers regarding gene therapy. The initial survey was an 8-item online survey administered by the National Fragile X Foundation (NFXF) to its broader community network, completed by 351 FXS community members. A follow-up survey was a 26-item instrument administered to 56 parents and caregivers recruited through clinics and the National Fragile X International Conference. Together, these surveys aim to characterize the current state of community readiness, identify specific educational gaps, clarify preferred channels for information delivery, and generate insights that can inform the design of future FXS gene therapy trials, informed consent processes, and regulatory strategy.

## 2 Materials and methods

### 2.1 Initial survey (survey one)

The initial survey, or Survey One, was an anonymous, cross-sectional online survey to characterize the FXS community’s knowledge, attitudes, and concerns regarding gene therapy (Supplementary File 1). The instrument comprised eight questions spanning multiple-choice, yes/no, ranking, and free-response formats and was designed to be completed in approximately 5 min. No demographic information was collected. Participants could skip any question. Recruitment was conducted via the NFXF website and members of the NFXF who had registered for research opportunities and agreed to be contacted via email. Eligible participants were English-speaking adults aged 18 or older who self-identified as members of the FXS community, including parents, caregivers, and adult individuals with FXS. A total of 351 responses were collected and analyzed. Descriptive statistics were used to summarize key themes across responses.

### 2.2 Survey two

Survey Two was a cross-sectional, anonymous survey study administered via REDCap v14.0.29 (Vanderbilt University, Nashville, TN). A 26-item instrument was developed by the research team, informed by prior published gene therapy perception studies (Maertens et al., 2022) and structured input from the NFXF Research Readiness Program (RRP), a collaborative initiative that facilitates FXS clinical and translational research through a review committee of FXS clinical and scientific experts (Supplementary File 2). The instrument underwent face validity review and cognitive pilot testing with a small panel of FXS caregivers and clinical professionals prior to finalization. The final instrument included demographic items, Likert-scale and multiple-choice questions assessing gene therapy attitudes and willingness, and true/false knowledge items assessing understanding of AAV-based gene therapy. A brief standardized description of AAV-based gene therapy was provided to all participants at the start of the survey to provide participants with a common informational framework; therefore, knowledge scores in Survey Two reflect performance after a brief educational exposure rather than pre-exposure baseline knowledge. The responses were elicited following a standardized, hypothetical description of gene therapy. The measured attitudes reflect participants’ responses to this framing and may not capture unprompted or pre-existing community attitudes. Participation was voluntary, no personally identifiable information was collected, and respondents could skip any question. Eligible participants were English-speaking caregivers of individuals diagnosed with FXS, aged 18 or older. Recruitment occurred both in-person at the National Fragile X International Conference and online via NFXF, email listserv, and the MyFXResearch portal. The study received a determination of human research exemption from the Institutional Review Board of the Icahn School of Medicine at Mount Sinai (STUDY-24-00750). Of 77 responses received, 56 surveys meeting a completion threshold of ≥ 90% of items were included in the analysis.

All data were analyzed using Microsoft Excel (Microsoft Corp., Redmond, WA) and SAS Studio v3.81 (SAS Institute Inc., Cary, NC). Descriptive statistics were used to summarize demographic characteristics and response distributions. The Kruskal-Wallis test was used to evaluate associations between caregiver education level and gene therapy knowledge scores, with a two-sided alpha of 0.05. Qualitative free-text responses were summarized thematically and visualized using a word frequency cloud generated via for illustrative purposes. For Survey Two, additional analyses were performed in Python (version 3.12; pandas, scipy, statsmodels, scikit-learn libraries). Spearman’s rank correlations were computed to assess associations between continuous predictor variables (caregiver age, FXS individual age, knowledge score, education level, total symptom severity) and attitude items (Att-1: hopefulness; Att-2: willingness to consider gene therapy) and among attitude items to assess internal consistency. Kruskal–Wallis tests were used to evaluate between-group differences in attitude scores across FXS age groups (children 0–12 years, adolescents 13–17 years, adults ≥ 18 years). To capture the full priority distribution beyond first-ranked responses, weighted scores were computed for concern and motivator items by assigning 3, 2, or 1 point to rank-1, rank-2, and rank-3 selections, respectively. A k-means clustering analysis (*k* = 3) was conducted to identify data-driven caregiver subgroups using attitude scores, knowledge score, and weighted concern and motivator features; features were standardized prior to clustering. The number of clusters (*k* = 3) was selected by maximizing the silhouette coefficient across *k* = 2 to *k* = 7 (optimal silhouette = 0.145 at *k* = 3). Cluster stability was assessed by bootstrapped resampling (200 iterations); the mean adjusted Rand index (ARI) between original and bootstrap cluster assignments was 0.47 (SD = 0.21), indicating moderate reproducibility. Given the modest silhouette coefficient and smallest cluster of *n* = 11, these subgroups are considered exploratory. All significance tests used a two-sided α = 0.05.

## 3 Results

### 3.1 Demographic characteristics

Survey One was completed by 351 members of the broader FXS community via the NFXF online platform. No demographic information was collected for Survey One, as the instrument was designed as a brief community pulse survey prioritizing response volume and accessibility over demographic characterization. Survey Two was completed by 56 caregivers. Caregiver age was available for 53 respondents and ranged from 22 to 73 years, and most were older than 40 years of age. Educational attainment data were available for 54 respondents; the majority held a college degree or higher (79.6%). The predominant caregiver relationship was mother (*n* = 47, 83.9%). Consistent with the X-linked inheritance pattern of FXS, the majority of individuals with FXS cared for were male (*n* = 47, 85.5%), with seven females (12.5%) and one respondent selecting “other” (1.8%). Full demographic characteristics are presented in Table 1. Duplicate participation across both surveys cannot be entirely excluded given the anonymous nature of both instruments, which is acknowledged as a limitation.

**TABLE 1:** Demographic characteristics of Survey Two respondents (FXS caregivers, *n* = 56).

Characteristic	n	%
Caregiver age (years), n = 53		
18–29	1	1.9%
30–39	9	17.0%
40–49	19	35.8%
50–59	17	32.1%
≥ 60	7	13.2%
Caregiver education, n = 54		
Less than high school	0	0%
High school diploma/equivalent	3	5.6%
Associate degree	8	14.8%
College degree (BA/BS)	22	40.7%
Post-graduate degree (MA/PhD)	21	38.9%
Caregiver relationship to individual with FXS, n = 56		
Mother	47	83.9%
Father	8	14.3%
Sibling	1	1.8%
Sex of individual with FXS, n = 56		
Male	47	85.5%
Female	7	12.5%
Other/prefer not to answer	2	3.6%
Age of individual with FXS (years), n = 54		
0–5	4	7.4%
6–12	11	20.4%
13–17	10	18.5%
18–29	16	29.6%
≥ 30	13	24.1%

Most caregivers were mothers (83.9%), aged 40 years or older (81.1%), and held a college or post-graduate degree (79.6%). Age and education data were available for 53 and 54 respondents, respectively; all other variables had complete data (*n* = 56). FXS, Fragile X syndrome.

### 3.2 Perspectives toward willingness to participate in gene therapy

**FIGURE 1:**
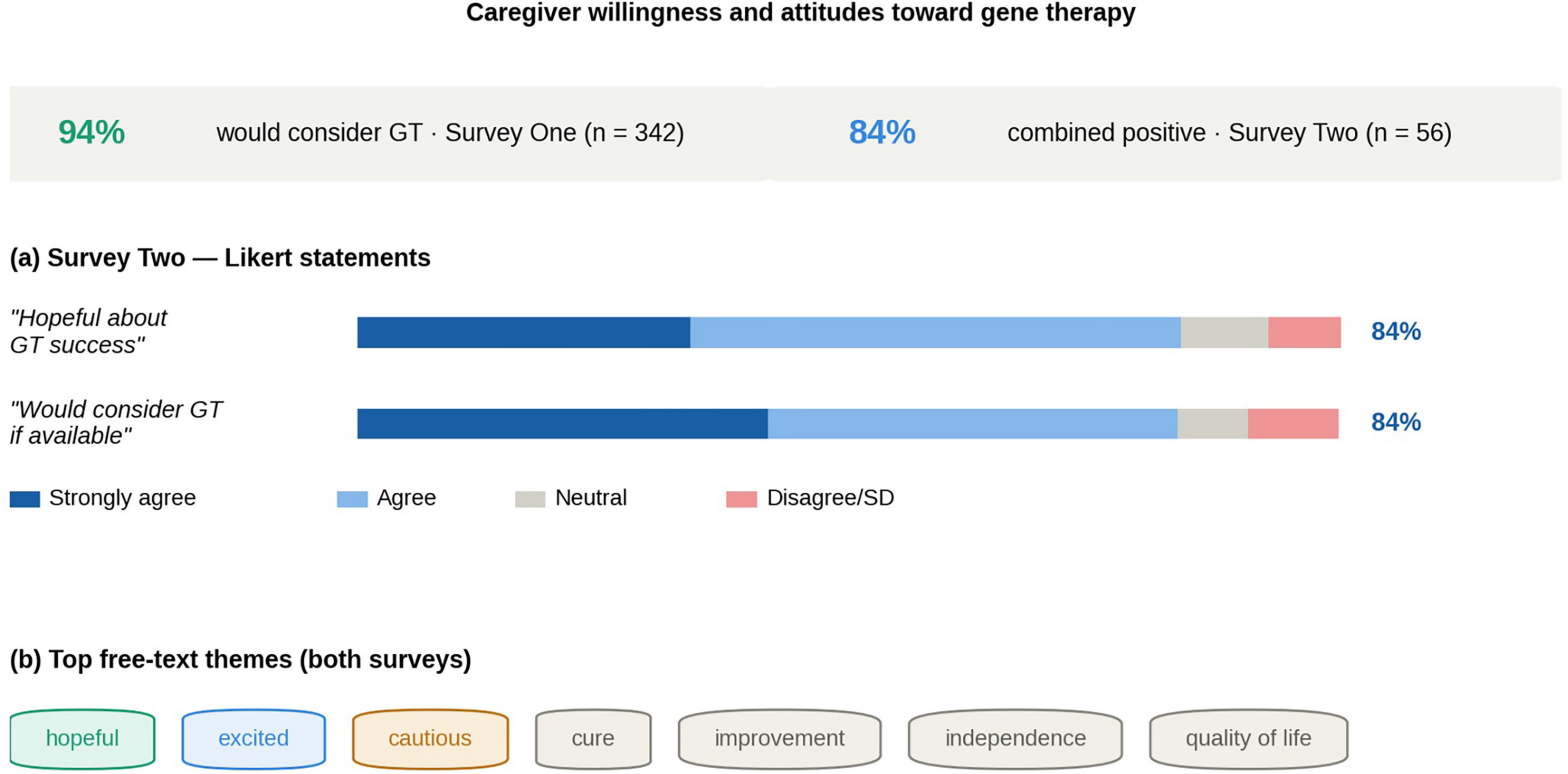
Caregiver willingness and attitudes toward gene therapy. 94% of caregivers (Survey One, *n* = 342) indicated they would consider gene therapy for FXS. In Survey Two (*n* = 56), 84% agreed or strongly agreed they were hopeful about gene therapy success (“Hopeful about GT success”) and 84% agreed or strongly agreed they would consider gene therapy if available (“Would consider GT if available”). **(a)** Survey Two Likert responses to two attitude statements (‘Hopeful about GT success’ and ‘Would consider GT if available’); **(b)** top free-text themes across both surveys, shown as tag labels.

In Survey One, respondents were asked directly: “Would you consider exploring gene therapy for FXS?” The response was strongly affirmative: 323 of 342 respondents with valid answers (94%) indicated yes, while only 19 (5.6%) indicated that they were not interested (Figure 1). In response to a free-text item asking participants to describe how they felt about gene therapy for FXS, responses were overwhelmingly positive in tone. The most frequently appearing terms included “hopeful,” “excited,” and “cautious,” reflecting a community that is enthusiastic while remaining attentive to risk.

Caregiver attitudes toward gene therapy were assessed in Survey Two using two Likert-scale statements administered after a standardized introductory description of AAV-based gene therapy. Responses to both statements were strongly positive. For Attitude Statement 1, “I am hopeful about the success of gene therapy to treat FXS,” 50.0% of respondents agreed and 33.9% strongly agreed, yielding a combined positive response rate of 84%. For Attitude Statement 2, “Gene therapy is something I would consider for the individual living with FXS that I care for if it were available,” 42% agreed and 42% strongly agreed, for a combined positive response rate of 84%. Neutral responses were 9% and 7% for Statements 1 and 2, respectively, and negative responses were minimal at 7% and 9%. Notably, the proportion of respondents who strongly agreed to Statement 2 (42%) equaled those who agreed, suggesting that caregiver willingness reflects conviction rather than passive openness. The combined positive rates were nearly identical across both statements, indicating internal consistency in caregiver attitudes (Figure 1). The word frequency distribution of free-text responses describing how participants felt about gene therapy for FXS is illustrated in Supplementary Figure 1.

Spearman’s rank correlations among the three Survey Two attitude items confirmed internal consistency: hopefulness (Att-1) and willingness to consider gene therapy (Att-2) were strongly correlated (rho = 0.523, *p* < 0.001), as were Att-1 and parental study design preference (Att-3; rho = 0.450, *p* = 0.001) and Att-2 and Att-3 (rho = 0.587, *p* < 0.001). Notably, concern orientation did not predict lower willingness: caregivers who weighted safety concerns most highly did not report significantly lower willingness to consider gene therapy (rho = −0.094, *p* = 0.495), indicating that the surveyed caregivers are motivated to pursue gene therapy despite their concerns, rather than deterred by them. Across both surveys, caregiver attitudes were consistently and strongly positive, though direct comparison should be interpreted cautiously given substantial differences in survey design, recruitment, and content between the two instruments. Taken together, the data suggest a consistent pattern: the surveyed caregivers demonstrate high motivation and broadly positive attitudes toward gene therapy.

### 3.3 Perspectives toward motivators to participate in gene therapy and symptom improvement priorities

**FIGURE 2:**
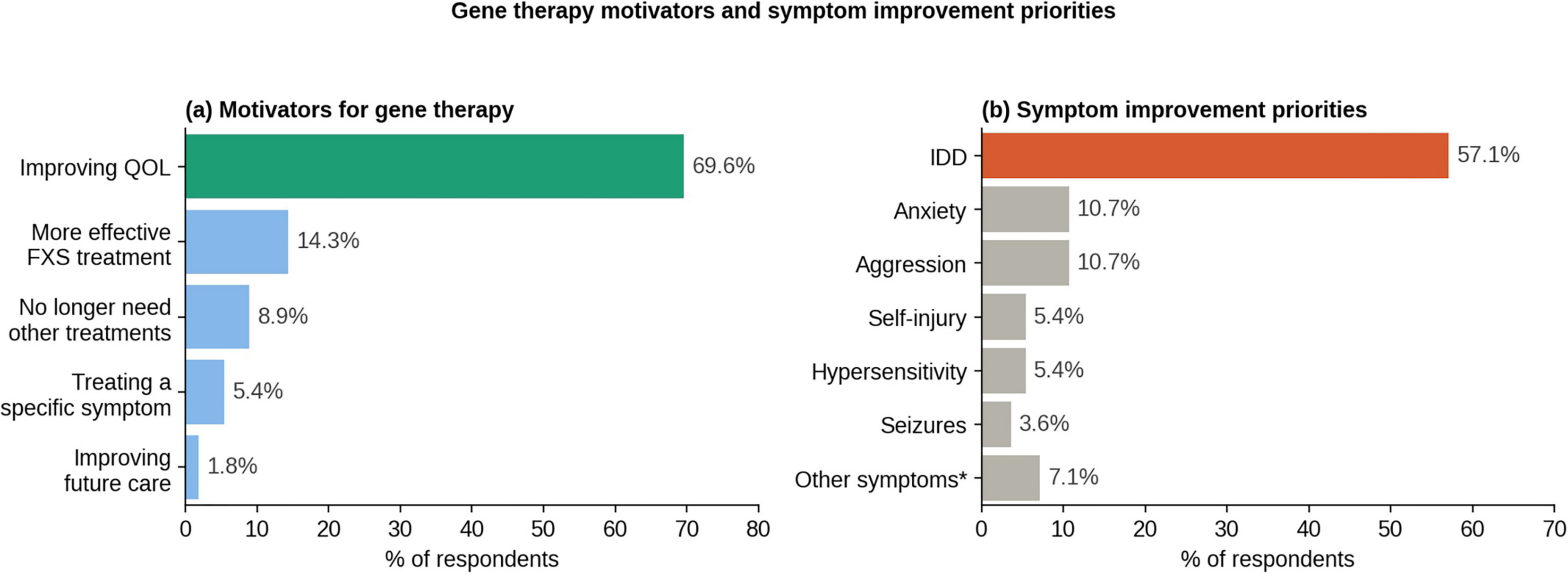
Gene therapy motivators and symptom improvement priorities among (FXS caregivers, *n* = 56). **(a)** Improving overall quality of life was the leading gene therapy motivator, selected as the top priority by 69.6% of respondents. **(b)** Intellectual and developmental disability (IDD) was the top-ranked symptom improvement target (57.1%), selected more than five times as often as the next-ranked symptoms (anxiety and aggression, 10.7% each). Values represent rank-1 selections only. QOL, quality of life; IDD, intellectual and developmental disability. *Hyperactivity, hyperarousal, attention problems, and irritability (1.8% each).

Participants in Survey Two were presented with six common motivators for pursuing gene therapy and asked to rank their top three, with rank 1 representing their most important motivator. Improving overall quality of life (QOL) for the individual living with FXS was the primary motivator (69.6%), selected by nearly seven in ten respondents as their top reason to pursue gene therapy. The need for more effective FXS treatment was the second most common first-ranked motivator (14.3%), followed by potential to no longer need other treatments (8.9%) and treating a particular symptom (5.4%) (Figure 2).

Participants were further asked to imagine that a treatment could improve up to five symptoms and to rank the top five they most wished a hypothetical gene therapy could address. Intellectual and developmental disability (IDD) was ranked as the primary symptom to improve by most respondents (*n* = 32, 57.1%), more than five times the frequency of the next-ranked symptoms. Anxiety and aggression each received six first-ranked responses (10.7%). Additional first-ranked symptom priorities included self-injury (5.4%), hypersensitivity (5.4%), seizures (3.6%), and 1.8% each for hyperactivity, hyperarousal, attention problems, and irritability. ASD features, depression, and sleep disorders received no first-ranked responses. The predominance of IDD, selected by more than half of all respondents, suggests that caregivers regard restoration of cognitive and learning ability as the most consequential potential benefit of gene therapy for FXS (Figure 2).

### 3.4 Perspectives toward concerns of gene therapy

Survey One respondents were presented with 12 potential gene therapy concerns, including side effects and risks, timeline to availability, treatment frequency, treatment delivery method, cost, availability, effectiveness, newness, trustworthiness of sponsors and pharmaceutical companies, ethical concerns, life after gene therapy, and other. Participants were asked to rank them in order of importance. The most frequently top-ranked concern was side effects and risks (*n* = 169, 52.5%), representing more than half of all first-ranked responses and more than 2.5 times the frequency of the next-ranked concern. Timeline to availability was second (*n* = 62, 19.6%), followed by effectiveness (*n* = 27, 8.5%). All remaining concerns each accounted for less than 8% of first-ranked responses. The full distribution is presented in Table 2.

**TABLE 2:** First-ranked gene therapy concerns among Survey One (NFXF community, *n* = 351) and Survey Two (FXS caregivers, *n* = 56) respondents.

Concern	Survey One (NFXF)		Survey Two (FXS caregivers)		Both
	*n*x	%	*n*	%	
Side effects / Risks	169	52.5%	26	46.4%	√
Timeline to availability	62	19.3%	–	–	S1
Effectiveness	27	8.4%	7	12.5%	√
Cost	–*	–*	7	12.5%	S2
Newness / Lack of long-term data	–*	–*	6	10.7%	S2
Life after gene therapy	–*	–*	3	5.4%	√
Unable to re-treat in the future	–†	–†	2	3.6%	S2
Time commitment / Travel	–†	–†	2	3.6%	S2
Availability	–*	–*	1	1.8%	√
Gene therapy composition	–†	–†	1	1.8%	S2
Other (free text: “safety”)	–*	–*	1	1.8%	√
Ethical concerns	–*	–*	0	0%	√
Treatment frequency	–*	–*	–†	–†	S1
Treatment delivery method	–*	–*	–†	–†	S1
Trustworthiness of sponsors	–*	–*	–†	–†	S1
Lack of diversity in clinical trials	–†	–†	0	0%	S2
Total respondents	322	100%	56	100%	–

Values represent first-ranked responses only (respondents’ single most prominent concern). √ = item present in both survey instruments. S1 = Survey One only; S2 = Survey Two only; no comparable data exist for the other survey. *Survey One frequencies for ranks 4–16 to be confirmed from raw data; top three values are as reported. † Item not included in that survey’s instrument. NFXF, National Fragile X Foundation; FXS, Fragile X syndrome.

Participants in Survey Two were presented with a similar list of 12 potential AAV gene therapy concerns, including cost, side effects and risks, effectiveness, newness and lack of long-term data, inability to receive gene therapy again if administered now, ethical concerns, availability, life after gene therapy, gene therapy composition, lack of diversity in clinical trials, time commitment and travel, and other. Participants were asked to rank their top three, with rank 1 representing their most prominent concern. Consistent with the initial survey, side effects and risks was the most frequently cited first-ranked concern (*n* = 26, 46.4%). Cost and effectiveness each accounted for the second tier (*n* = 7 each, 12.5%), followed by newness and lack of long-term human data (*n* = 6, 10.7%). The remainder of first-ranked responses were distributed across life after gene therapy (*n* = 3, 5.4%), inability to re-treat in the future (*n* = 2, 3.6%), time commitment and travel (*n* = 2, 3.6%), availability (*n* = 1, 1.8%), gene therapy composition (*n* = 1, 1.8%), and other (*n* = 1, 1.8%; free-text: “safety,” which conceptually overlaps with the leading concern). Ethical concerns and lack of diversity in clinical trials received no first-ranked responses, suggesting these were not regarded as primary barriers to gene therapy acceptance. The full distribution is presented in Table 2.

Across both surveys, side effects and risks emerged as the dominant primary concern, representing 52.5% and 46.4% of first-ranked responses in Survey One and Survey Two, respectively. This convergence across two sequential surveys reinforces the centrality of safety in the FXS community’s consideration of gene therapy. Weighted scoring across all top-3 concern selections (rank 1 = 3 pts, rank 2 = 2 pts, rank 3 = 1 pt) confirmed and strengthened this finding: side effects and risks accumulated a weighted score of 116, more than twice the next-ranked concern (effectiveness: 41), followed by newness/lack of long-term data (36) and cost (32). This graded priority distribution demonstrates that the primacy of safety concerns is not a threshold effect but reflects a robust preference across the full range of top-3 rankings. Similarly, weighted motivator scoring confirmed that improving overall quality of life was the leading motivator (weighted score: 141), more than twice the score of the next-ranked motivator (need for more effective FXS treatment: 59), reinforcing the centrality of functional and quality-of-life outcomes across all ranking positions (Supplementary Table 1).

### 3.5 Caregiver knowledge toward gene therapy

**FIGURE 3:**
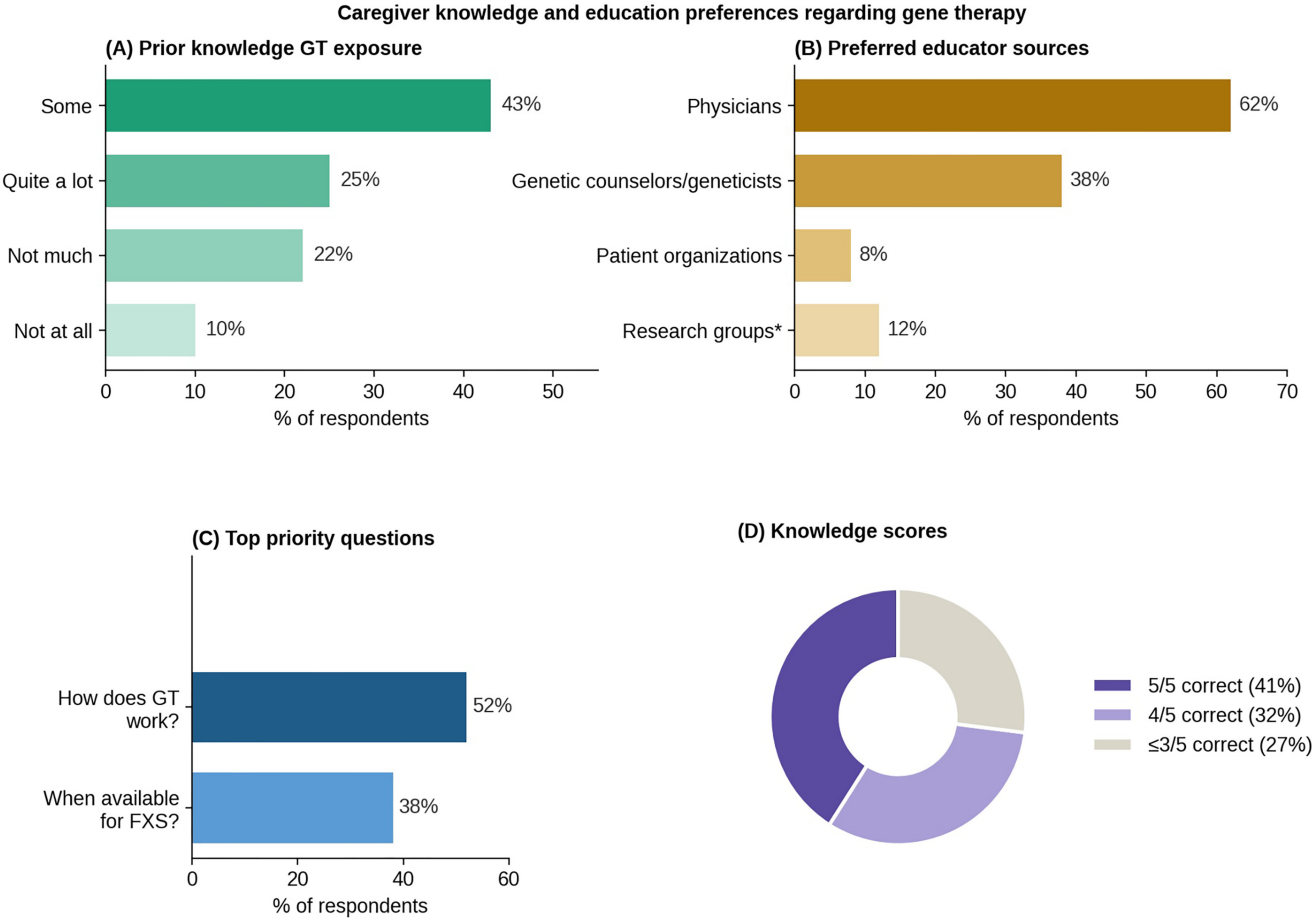
Caregiver knowledge and education preferences regarding gene therapy. **(A)** Self-reported prior gene therapy knowledge exposure, Survey One: most respondents reported “some” prior knowledge (43%), while 10% reported none at all. **(B)** Preferred sources for gene therapy education, Survey Two: physicians were ranked first by 62% of respondents, followed by genetic counselors/geneticists (38%); *“research groups” identified via free-text response. **(C)** Two most frequently top-ranked priority questions about gene therapy, Survey One: “How does GT work?” (52%) and “When will it be available for FXS?” (38%). **(D)** True/false knowledge score distribution following standardized AAV gene therapy introduction, Survey Two: 73% of respondents scored ≥ 4/5 correct. GT, gene therapy; AAV, adeno-associated virus; NFXF, National Fragile X Foundation.

In Survey One, to begin assessing community knowledge, participants were first broadly asked how much they had heard/read about genetic testing. Ninety-one respondents (25%) answered quite a lot, 162 respondents (43%) said some, 72 respondents (22%) said not much, and 25 respondents (10%) answered not at all (Figure 3A). Most survey respondents had previous knowledge of gene therapy before taking the survey. However, while participants had some knowledge regarding gene therapy, most respondents (*n* = 292, 84.2%) were not familiar with the different types of gene therapy currently available, and only 55 respondents (22.3%) were familiar with this information. Of the respondents, 62% ranked physicians as the top group to inform them, followed by genetic counselors and geneticists (38%) (Figure 3B). Other respondents also used the free response option to identify “research groups” as another avenue for further education about gene therapy. Survey respondents were asked to rank in order the most important questions they wanted answered about gene therapy. Options included how gene therapy works, how long developing gene therapy takes, how long gene therapy effects last (is it a cure?), when gene therapy will be available for FXS, who will be able to participate in gene therapy studies (e.g., age, sex, diagnosis), availability/insurance coverage, risks, whether effectiveness of gene therapy will differ depending on the individual (e.g., mosaicism, methylation status, sex), what gene therapy is made of (cells, medications, viral vectors), and gene modification strategies. Of these 11 potential options, how gene therapy works was most frequently rated as the top question by 162 participants (52%), followed by when it will be available for FXS by 103 participants (38%) (Figure 3C). The third most popular question was how long gene therapy effects last (is it a cure?). Participants also had the option to provide a free-text response regarding any additional areas of concern they wanted clarified before considering participation in future gene therapy research. Common themes that arose from the 257 responses were side effects/risks (*n* = 62), safety (*n* = 18), effectiveness (*n* = 18), and cost (*n* = 13).

Introductory information on AAV-based gene therapy was provided at the start of Survey Two. Survey respondents answered five true or false knowledge questions about gene therapy to assess knowledge following the standardized educational introduction provided at the start of Survey Two. Most respondents (*n* = 23, 41%) answered all five questions correctly, and 18 respondents (32%) answered four out of five questions correctly (Figure 3D). The most commonly missed question was Question 3, which stated that “Gene therapy can be given to someone more than once.” The correct answer was false, as the survey questions were all regarding AAV gene therapy. Only 49% of participants correctly answered this question, indicating that most respondents incorrectly thought that AAV gene therapy could be given more than once. However, we acknowledge that some respondents may have been answering with a broader understanding of gene therapy. Other respondents also used the free response option to identify “research groups” as another avenue for further education about gene therapy.

Participants had the opportunity to ask questions they had regarding gene therapy. Of respondents, 15 left free-response questions, which revealed three general themes: effectiveness of treatment, availability/timeline of gene therapy, and side effects/risks of gene therapy. For example, one participant asked, “Would he be cured with no anxiety, aggression, behavior. Would he be able to learn things that he was unable to grasp before.” For the theme of availability/timeline of gene therapy, another participant asked “When????” and for the theme of side effects/risks, a participant asked, “How would you support the participants post-trial or in the event of side effects?” These questions revealed information gaps within the community and areas for further education.

### 3.6 Statistical associations between caregiver characteristics, FXS individual Age, and gene therapy attitudes

Kruskal-Wallis testing showed that caregiver education level did not impact gene therapy knowledge scores (*p* = 0.16). Additional Spearman’s rank correlations confirmed that neither knowledge score nor caregiver education was significantly associated with willingness to consider gene therapy (rho = −0.085, *p* = 0.541 and rho = 0.006, *p* = 0.968, respectively), and total symptom severity was similarly not associated with attitudes (all *p* > 0.49), indicating that willingness is distributed broadly across the caregiver population regardless of background or clinical severity.

Age of the individual living with FXS was significantly and inversely associated with caregiver willingness to consider gene therapy (Spearman rho = −0.324, *p* = 0.017) and with hopefulness about gene therapy success (rho = −0.298, *p* = 0.028). When stratified by developmental stage, attitude scores did not differ significantly across FXS age groups in Kruskal–Wallis tests: hopefulness (Att-1: H = 5.424, df = 2, *p* = 0.066, ε^2^ = 0.067) and willingness (Att-2: H = 5.621, df = 2, *p* = 0.060, ε^2^ = 0.071) showed trends toward higher scores among caregivers of younger individuals, but no pairwise comparison reached significance after Bonferroni correction (children 0–12, *n* = 15; adolescents 13–17, *n* = 10; adults ≥ 18, *n* = 29; all Dunn adjusted *p* > 0.09). In contrast, caregiver preference for study design input (Att-3) differed significantly across groups (H = 10.303, df = 2, p = 0.006, ε^2^ = 0.163): caregivers of children (0–12 years; median = 5.0, IQR 4.0–5.0) scored significantly higher than caregivers of adults ( ≥ 18 years; median = 3.0, IQR 3.0–4.0; Dunn adjusted *p* = 0.010), while caregivers of adolescents (13–17 years; median = 3.5, IQR 3.0–4.0) did not differ significantly from either group (all Dunn adjusted *p* > 0.07). Older caregiver age was also inversely correlated with willingness to consider gene therapy in bivariate analysis (rho = −0.298, *p* = 0.030), while caregiver age was not associated with hopefulness (rho = −0.008, *p* = 0.955) or knowledge score (rho = 0.121, *p* = 0.393).

An exploratory k-means clustering analysis (*k* = 3; *n* = 54 with complete data, silhouette coefficient = 0.145; bootstrap ARI = 0.47 ± 0.21) identified three caregiver subgroups: (1) Cautious/Experienced caregivers (*n* = 22) with moderate attitudes, higher knowledge scores, and the oldest mean caregiver and FXS individual ages (53.8 and 23.0 years, respectively); (2) Enthusiastic Engagers (*n* = 21) with the strongest attitudes across all items (mean Att-2 = 4.86) and the youngest mean ages (45.2 and 15.2 years); and (3) a Moderate/QOL-focused group (*n* = 11) with positive attitudes and the highest knowledge scores. Clusters differed significantly on caregiver age (H = 9.112, *p* = 0.011) and FXS individual age (H = 7.190, *p* = 0.027), but not on education or symptom severity (both *p* > 0.40) (Table 3). Given the limited sample size, these subgroup profiles should be interpreted as hypothesis-generating rather than definitive.

**TABLE 3:** Caregiver subgroup profiles identified by k-means clustering (*k* = 3; *n* = 54 with complete data), Survey Two.

Characteristic	Cluster 1 cautious/experienced (*n* = 22)	Cluster 2 enthusiastic engagers (*n* = 21)	Cluster 3 moderate/QOL-focused (*n* = 11)	H	*p*
Age (years), mean ± SD					
Caregiver age	53.8 ± 11.0	45.2 ± 10.2	55.8 ± 10.9	9.112	0.011
FXS individual age	23.0 ± 10.9	15.2 ± 10.3	22.7 ± 11.1	7.190	0.027
Caregiver education, median (scale 1–6)					
Education level	4.0	4.0	4.0	1.793	0.408
FXS symptom severity					
Total severity score, mean	17.4	17.8	18.5	0.522	0.770
Attitude scores, mean (scale 1–5)					
Att-1: Hopeful about GT success	3.86	4.67	4.09	–	–
Att-2: Would consider GT	3.64	4.86	4.27	–	–
Att-3: Study design preference	3.18	4.52	3.55	–	–
Knowledge					
Knowledge score (0–5), mean	4.14	3.95	4.27	–	–

H = Kruskal–Wallis statistic. Att-1 = “I am hopeful about the success of gene therapy to treat FXS.” Att-2 = “Gene therapy is something I would consider for the individual living with FXS that I care for if it were available.” Att-3 = “I would be more likely to enroll in gene therapy trials if researchers considered my preference about the study design.” GT, gene therapy; FXS, Fragile X syndrome; QOL, quality of life; SD, standard deviation. Education coded 1–6 ( = < high school, 4 = college degree, 5 = post-graduate). Significance level: α = 0.05. Bold *p*-values indicate statistical significance. K-means clustering (*k* = 3) applied to attitude scores, knowledge score, and weighted concern/motivator features (*n* = 54). Features z-score standardized prior to clustering. Kruskal–Wallis tests assessed between-cluster differences; — denotes clustering input variables. Cluster 1 (Cautious/Experienced): moderate willingness, older caregivers of adults with FXS. Cluster 2 (Enthusiastic Engagers): highest willingness and hopefulness, youngest caregivers and FXS individuals. Cluster 3 (Moderate/QOL-Focused): positive attitudes, highest knowledge score. Att-1 = “Hopeful about GT success.” Att-2 = “Would consider GT if available.” Att-3 = “Would enroll if study design considered my preference.” GT = gene therapy; FXS = Fragile X syndrome; QOL = quality of life; SD = standard deviation.

Taken together, these findings point to a community that is engaged and motivated to learn, but that requires structured, clinician-led education addressing the mechanism of AAV gene therapy, as well as realistic timelines, risk profiles, and individual eligibility. These priorities may inform the design of pre-trial community education programs and informed consent materials for future FXS gene therapy studies.

## 4 Discussion

This study presents findings from two sequential cross-sectional surveys, complementing a concurrent preliminary study (Eley et al., 2025). This study was conducted at a critical juncture in the field, as AAV-mediated gene therapy transitions from preclinical promise to realistic near-term clinical development. Together, these surveys captured the knowledge, attitudes, concerns, and information preferences of 407 individuals connected to the FXS community. This study provides actionable data to inform trial design, informed consent processes, and community education strategies.

The most striking and consistent finding across both surveys was the strongly positive attitude of FXS caregivers toward gene therapy. In both surveys, the great majority of respondents expressed positive attitudes toward gene therapy. In Survey One, 94% of community members indicated they would consider gene therapy (323/342 valid responses). In Survey Two, 84% of caregivers agreed or strongly agreed they were hopeful about gene therapy success, and 84% indicated they would consider gene therapy if available. These rates are notably high and clinically meaningful, particularly given that FXS is a non-lethal condition with a normal life expectancy, a context in which one might expect greater caregiver hesitancy when weighing the risks of an investigational intervention against the absence of life-threatening urgency. The parent/caregiver willingness rates to participate are comparable to those reported in communities where gene therapy addresses progressive or life-limiting conditions such as Duchenne muscular dystrophy and Friedreich ataxia (Landrum Peay et al., 2019; Trantham et al., 2023), suggesting that caregiver motivation in FXS is driven by the profound functional impact of the disorder rather than existential urgency alone (Kaufmann et al., 2024). Regardless, both surveys converge on a consistent message: the surveyed caregivers report high motivation and broadly positive attitudes toward gene therapy, though these findings reflect the perspectives of this surveyed sample and may not be generalized to all FXS caregivers.

On the motivator and treatment priority side, improving overall quality of life was the leading motivator (69.6%) and intellectual and developmental disability (IDD) was the top symptom priority (57.1%) which was selected by more than five times as many respondents as the next-ranked symptoms, anxiety and aggression (10.7% each) (Lozano et al., 2022). These data reflect what surveyed respondents valued most highly and may contribute to patient-preference discussions relevant to endpoint planning. Formal endpoint selection for future FXS gene therapy trials will require developmental validity assessment, clinical feasibility analysis, regulatory considerations, and broader community input beyond the scope of the present surveys. Side effects and risks was the dominant top-ranked concern across both surveys, selected by 46.4% and 52.5% of respondents. This finding is both robust and actionable. Informed consent materials for future FXS gene therapy trials must devote particular attention to transparent communication of the safety profile, adverse event monitoring procedures, and post-trial support. The convergence of this finding across two sequential surveys reinforces the centrality of safety concerns in the community’s consideration of gene therapy.

Both surveys identified a consistent divide between general awareness and specific mechanistic knowledge. In Survey One, 62.3% of respondents had prior familiarity with gene therapy, yet only 15.9% were familiar with the different types available, a gap of nearly 50 percentage points. In Survey Two, overall knowledge performance was high after receiving standardized introductory information (73.2% scoring ≥ 4/5), yet a critical misconception persisted: 50.9% of respondents incorrectly believed AAV gene therapy can be administered more than once. The top-ranked priority questions in Survey One — how gene therapy works, when it will be available for FXS, and how long effects last — together define the core curriculum needed before any FXS gene therapy trial. Physicians (53.6%) were identified as the preferred educational sources, highlighting the central role of FXS clinicians in preparing the community for informed trial participation. Notably, Kruskal-Wallis analysis found no association between caregiver education level and knowledge score (*p* = 0.16), indicating that these gaps are universal across the caregiver population and cannot be addressed by targeting specific families alone (Shuleski et al., 2022).

An interesting finding from the present study is the significant inverse association between age of the individual living with FXS and caregiver willingness to consider gene therapy. Caregivers of younger children (0–12 years) reported markedly higher willingness (mean 4.60/5) than caregivers of adolescents (4.31) or adults ≥ 18 years (4.00; Kruskal–Wallis *p* = 0.037), and FXS individual age was inversely correlated with both willingness (rho = −0.324, *p* = 0.017) and hopefulness (rho = −0.298, *p* = 0.028). Older caregiver age was also inversely correlated with willingness to consider gene therapy in bivariate analysis (rho = −0.298, *p* = 0.030). These findings likely reflect a developmental-window effect: caregivers of younger individuals may perceive greater potential benefit from early FMRP restoration, given the known importance of early synaptic development for cognitive outcomes in FXS, and may have had less time to accumulate experience with failed treatment attempts. Conversely, caregivers of adults with FXS may have adjusted family expectations and daily routines around existing management strategies, which could temper enthusiasm for an investigational intervention. This age gradient has direct implications for clinical trial recruitment and consent: families of younger individuals with FXS are likely to be the most motivated early adopters, and consent materials and community engagement efforts should be calibrated to their priorities. A complementary k-means subgroup analysis identified three distinct caregiver profiles: Cautious/Experienced (*n* = 22), Enthusiastic Engagers (*n* = 21), and Moderate/QOL-focused (*n* = 11), This subgroups differed significantly on caregiver age (H = 9.112, *p* = 0.011) and FXS individual age (H = 7.190, *p* = 0.027) but not on education or symptom severity, further supporting age as the primary driver of heterogeneity in caregiver attitudes toward gene therapy in this sample.

Both surveys used convenience sampling through NFXF channels and the National Fragile X Conference, introducing selection bias toward families already engaged in FXS advocacy and research. The Survey Two sample was predominantly female (83.9% mothers) and highly educated (79.6% college or postgraduate degree). Both surveys were conducted in English only, excluding non-English-speaking families who may hold different perspectives. Therefore, our findings may not fully represent caregivers with lower educational attainment, fathers, non-parent caregivers, or families with less access to specialty care or advocacy networks. Survey One collected no demographic information, which substantially limits our ability to assess the representativeness of those respondents; it is therefore unknown whether they are representative of the broader FXS caregiver population. The two surveys used different instruments, eligibility criteria, and recruitment contexts and are not directly comparable; convergent findings are presented as complementary rather than confirmatory. Survey Two’s sample size limits power for subgroup analyses. Survey Two also had more items and longer surveys consistently produce lower response and completion rates in the survey literature, which likely contributed to its smaller sample. The absence of a validated gene therapy attitude instrument for rare disease caregivers constrains direct comparison with other published studies.

In summary, the surveyed FXS caregivers report high motivation and broadly positive attitudes toward gene therapy, with safety as their foremost concern and improvement in cognitive function and quality of life as their primary goals. Specific knowledge gaps require targeted, clinician-led education prior to trial initiation. These findings represent the perspectives of surveyed caregivers and should be interpreted with appropriate caution regarding generalizability to the broader FXS community. They may nonetheless inform the design of FXS gene therapy trials, the development of informed consent materials, and community education programs, and provide a foundation for ongoing community engagement as the field advances toward first-in-human studies. These insights may also support future gene therapy trial design, consent processes, and pre-trial planning in a patient-centered manner.

## Data Availability

The raw data supporting the conclusions of this article will be made available by the authors, without undue reservation.

## Supplementary material

Supplementary Table 1 Weighted priority scores for gene therapy concerns and motivators across all top-3 selections.

Supplementary Figure 1 The word frequency distribution of free-text responses describing how participants felt about gene therapy for FXS.

Supplementary File 1 PDF of the Survey 1.

Supplementary File 2 PDF of the Survey 2.
