# Supplementary material for: Knowledge, attitudes, and perspectives on gene therapy for Fragile X syndrome: results from two community and caregiver surveys
Source: Front Mol Neurosci. 2026 Jul 29;19:1912516. doi: 10.3389/fnmol.2026.1912516 (PMC13461620; doi:10.3389/fnmol.2026.1912516)
Supplement: Supplementary file 1 [file Data_Sheet_1.pdf]

Caregiver Perspectives on Gene Therapy for Fragile X Syndrome

Thank you for your interest in this research. Please complete the survey below.

Thank you!

Study Title: Caregiver Perspectives on Gene Therapy for Fragile X Syndrome
Principal Investigator (Lead Researcher): Vivian Chen, BS
Physical Address: 1428 Madison Avenue Atran Building, 1st Floor, NY, NY 10029
Mailing Address: One Gustave L Levy Place Box 1497, NY, NY 10029
Phone: (724) 799-4960

The purpose of this research study is to better understand the knowledge, attitudes, and perspectives towards potential gene therapy within the Fragile X Syndrome (FXS) community. This research also aims to gather information for design and implementation of future gene therapy clinical trials. You are being asked to take part in this research study because you are a caregiver for an individual living with FXS.

Participation in this research study is voluntary. You can agree to join or not. You can also say yes now and change your mind later. Deciding not to be in the research study, now or later, will not affect your ability to receive medical care at Mount Sinai.

If you choose to take part, you will be asked to complete the following anonymous survey. Your participation in this research study is expected to last 20 minutes. There are at least 25 people expected to take part in this research study.

You can choose not to answer any question you do not wish to answer. You can also choose to stop taking the survey at any time. You must be at least 18 years old to participate. If you are younger than 18 years old, please stop now.

The possible risks to you in taking part in this research are:
• Feeling uncomfortable
• Risk of loss of private information. This risk always exists, but there are procedures in place to minimize the risk. This survey is anonymous.

The possible benefits to you for taking part in this research are:
• Having your opinions shared with the research community before gene therapy clinical trials move forward
• Potentially informing the design of future clinical trials for FXS gene therapy

To protect your identity as a research subject, no identifiable information will be collected. In any publication about this research, your name or other private information will not be used.

If you have any questions about this research, please contact the Lead Researcher at (724) 799-4960. You can also call the Program for the Protection of Human Subjects Office at (212) 824-8200.

---

1 Do you care for an individual with FXS?
○ Yes
○ No

---

2 Has their FXS diagnosis been genetically confirmed (FMR1 full mutation > 200 repeats)?
○ Yes
○ No/I'm not sure

If you answered “No” to either of these questions, please stop now. To be eligible to participate in this survey, you must care for someone with FXS. Thank you for your time.

The purpose of this survey is to better understand your perspectives on gene therapy to treat FXS.

The U.S. Food and Drug Administration (FDA), defines gene therapy as a "method of modifying a person's genes to cure or treat a disease." Gene therapy has been approved by the FDA to treat other genetic conditions in humans. Research has been promising for gene therapy to treat FXS. However, given that this is a complex area of medicine, we want to hear your thoughts!

While there are many medications and interventions to help manage FXS, there is currently no treatment that targets the underlying genetic cause of FXS. In FXS, the Fragile X gene called FMR1 is affected. The FMR1 gene is unable to make the Fragile X protein called FMRP, which causes the symptoms of FXS.

Gene therapy aims to fix the FMR1 gene so the body can make FMRP again and successfully treat FXS. This could potentially mean no longer needing other forms of treatment as the underlying cause of FXS would be addressed.

Typically, gene therapy uses a vector or "vehicle" to deliver the healthy copy of the gene into the body. A vector is a modified virus that should not make you sick and will carry the healthy FMR1 gene into your body. While gene therapy is very well studied before it can be given to humans, there is always a risk of unknown side effects and immune responses.

Also, it is unknown how long the effects of gene therapy will last. However, with the current technology, gene therapy cannot be given more than once to an individual. If gene therapy were given more than once, the body would now recognize the vector and attack it as a defense mechanism. Therefore, it is important to figure out the optimal time to give an individual gene therapy.

As you can see, gene therapy is a complex topic, and we look forward to hearing your perspectives!

Please answer the questions below as accurately as possible. If you do not wish to answer any question, please feel free to skip it.

---

3 What is your current age, in years?

____

---

4 What is your highest level of education? If you are currently completing a degree, please select that option.

- ◯ Less than high school
- ◯ High school diploma or equivalent
- ◯ Associate degree (e.g. AA, AS)
- ◯ College degree (e.g. BS, BA)
- ◯ Post-graduate degree (e.g. MA, PhD)
- ◯ Prefer not to answer

---

5 What is your relationship to the individual living with FXS?

- ◯ Mother
- ◯ Father
- ◯ Sibling
- ◯ Other

Please specify other caregiver type:

____

---

6 What is the current age of the individual living with FXS, in years? If they are less than 1 years old, please enter 0.

____

---

7 What is the biological sex of the individual living with FXS?

- ◯ Male
- ◯ Female
- ◯ Intersex
- ◯ Other
- ◯ Prefer not to answer

Please specify other biological sex:

____

Does the individual living with FXS have any of the following? If so, please indicate the severity.

	None	Very Mild	Mild	Moderate	Severe
Intellectual Developmental Disability (IDD)	○	○	○	○	○
Autism Spectrum Disorder (ASD)	○	○	○	○	○
Depression and/or anxiety	○	○	○	○	○
Seizures	○	○	○	○	○
ADD/ADHD	○	○	○	○	○
Aggression and/or self-injury	○	○	○	○	○

Below is a list of FXS symptoms. Imagine a treatment could improve up to 5 of these symptoms. Please rank the top 5 symptoms you would want to improve, with 1st being the symptom you would most want to improve.

	1st	2nd	3rd	4th	5th
Intellectual Developmental Disability (IDD)	○	○	○	○	○
Autism Spectrum Disorder (ASD)	○	○	○	○	○
Depression	○	○	○	○	○
Anxiety	○	○	○	○	○
Seizures	○	○	○	○	○
Hypersensitivity / Overreaction to stimuli	○	○	○	○	○
Hyperactivity	○	○	○	○	○
Hyperarousal	○	○	○	○	○
Attention problems	○	○	○	○	○
Irritability	○	○	○	○	○
Aggression	○	○	○	○	○
Self-injury	○	○	○	○	○
Sleep disorders (for example: insomnia or sleep apnea)	○	○	○	○	○
None	○	○	○	○	○
Other	○	○	○	○	○

Please specify what other symptom you would want treatment to improve: ________________________________

The following questions will ask about how effective you think current treatments for FXS are in your experience:

	Not effective	Minimally effective	Somewhat effective	Very effective	Not sure
In your experience, how would you rate the effectiveness of medications to treat symptoms of FXS?	○	○	○	○	○
In your experience, how would you rate the effectiveness of non-medication interventions (for example: speech therapy, occupational therapy, applied behavior analysis) to manage symptoms of FXS?	○	○	○	○	○

The following questions will ask you about your attitudes on gene therapy.

	Strongly Disagree	Disagree	Neither Agree nor Disagree	Agree	Strongly Agree
I am hopeful about the success of gene therapy to treat FXS.	○	○	○	○	○
Gene therapy is something I would consider for the individual living with FXS that I care for if it were available.	○	○	○	○	○
I would be more likely to enroll in gene therapy trials if researchers considered my preference about the study design.	○	○	○	○	○

Before gene therapy for FXS becomes available as a treatment option, clinical trials will be necessary. A clinical trial is a research study in which human subjects are randomly assigned to an intervention, such as gene therapy, to evaluate the effects of the interventions.

If you or the individual living with FXS have participated in a clinical trial before, how would you rate your experience?

- ◯ Positive
- ◯ Neutral
- ◯ Negative
- ◯ We have not participated in a clinical trial before

Which of the following do you think is most important when considering who to enroll in gene therapy clinical trials?

- ◯ Age of the individual
- ◯ Severity of their symptoms
- ◯ Sex of the individual
- ◯ Mutation status (for example: full mutation, mosaic)
- ◯ Other

Please specify other considerations: ______________________________

Below is a list of common concerns regarding gene therapy. Please rank your top 3 concerns, with 1st being your most prominent concern.

	1st concern	2nd concern	3rd concern
Cost	○	○	○
Side effects / Risks	○	○	○
Effectiveness of treatment	○	○	○
Newness (no long term data in humans yet)	○	○	○
Not being able to get gene therapy in the future if it is given now	○	○	○
Ethical concerns (for example: religion, culture, consent)	○	○	○
Availability (how easy it is to access gene therapy)	○	○	○
Life after gene therapy	○	○	○
What the gene therapy is made of (for example: viral vector)	○	○	○
Lack of diversity in gene therapy clinical trials	○	○	○
Time commitment/travel	○	○	○
Other	○	○	○

Please specify what other concern you have regarding gene therapy: ______________________________

Below is a list of common motivators for gene therapy. Please rank your top 3 motivators for pursuing gene therapy, with 1st being your most prominent motivator

	1st motivator	2nd motivator	3rd motivator
Need for a more effective FXS treatment	○	○	○
Potential to no longer need other medications and interventions	○	○	○
Treating a particular symptom of FXS (for example: seizures, irritability)	○	○	○
Improving overall quality of life for the individual living with FXS	○	○	○
Improving care for future individuals with FXS	○	○	○
Other	○	○	○

Please specify what other motivator you have for pursuing gene therapy: ______________________________

Of the following, please rank who you think would be best to provide information on gene therapy clinical trials with 1 being the most preferred. Please note, medical professionals should ultimately be consulted on gene therapy.

	1	2	3	4	5
Doctors	○	○	○	○	○
Nurses	○	○	○	○	○
Genetic counselors/geneticists	○	○	○	○	○
Patient organizations	○	○	○	○	○
Other, please specify in the next question	○	○	○	○	○

Please specify, if any, what other sources you would prefer to receive information regarding gene therapy. ______________________

The following questions will ask you about your knowledge of gene therapy. Please select True or False for the following statements to the best of your ability:

Gene therapy typically involves a viral vector.	◯ True<br>◯ False
Gene therapy to treat FXS is already available.	◯ True<br>◯ False
Gene therapy can be given to someone more than once.	◯ True<br>◯ False
Because gene therapy is so well researched before it's given to humans, there are no known risks associated with gene therapy.	◯ True<br>◯ False
Gene therapy aims to treat the underlying cause of genetic conditions like FXS.	◯ True<br>◯ False
What are 3 words you would use to describe how you feel about gene therapy to treat FXS?	____________________________<br>(Please list 3 words.)
What are some questions you have about gene therapy?	◯ I have no questions at this time.<br>◯ I have questions.
Please write your questions about gene therapy here:	__________________________________
