# Supplementary material for: Knowledge, attitudes, and perspectives on gene therapy for Fragile X syndrome: results from two community and caregiver surveys
Source: Front Mol Neurosci. 2026 Jul 29;19:1912516. doi: 10.3389/fnmol.2026.1912516 (PMC13461620; doi:10.3389/fnmol.2026.1912516)
Supplement: Supplementary file 2 [file Data_Sheet_2.pdf]

1. How much have you heard or read about genetic testing?

- ◯ Quite a lot
- ◯ Some
- ◯ Not much
- ◯ Nothing at all

2. Did you know about gene therapy before taking this survey?

- ◯ Yes
- ◯ No

3. Are you familiar with the different types of gene therapy out there?

- ◯ Yes
- ◯ No

4. What do you want to know about gene therapy the most? Rank in order of most important to least important by dragging the rows.

- ☐ How does gene therapy work?
- ☐ How long does developing gene therapy take?
- ☐ How long do gene therapy effects last? (Is it a cure?)
- ☐ When will gene therapy be available for Fragile X?
- ☐ Who will be able to participate in gene therapy studies (i.e. age, sex, diagnosis, etc.)?
- ☐ Who will be able to have gene therapy (i.e. age, sex, diagnosis, etc.)?
- ☐ Availability/insurance coverage?
- ☐ Risks (i.e. mild side effects, death, unforeseen risks)?
- ☐ Will the effectiveness of gene therapy differ depending on the individual i.e. mosaicism, methylation status, sex?
- ☐ What is gene therapy made of (cells, medications, viral vectors, etc.)?
- ☐ What are gene modification strategies?

5. What are you most concerned about with gene therapy? Rank in order of most important to least important by dragging the rows.

- [ ] Side effects / Risks (i.e. mild side effects, death, unforeseen risks)?
- [ ] Timeline (when will this be available)
- [ ] Treatment frequency (i.e. 1x only, 3x per year, etc.)
- [ ] Treatment delivery (how the gene therapy administered to the individual i.e. infusion, medication, etc.)
- [ ] Cost
- [ ] Availability
- [ ] Effectiveness
- [ ] Newness
- [ ] Trustworthiness of sponsors/pharmaceutical companies
- [ ] Ethical concerns (i.e. religion, culture, consent (parent vs. individual receiving therapy))
- [ ] Life after gene therapy
- [ ] Other

6. Would you consider exploring gene therapy for yourself/your loved one with FXS?

- ○ Yes
- ○ No

7. What are you most looking forward to with advances in gene therapies?

[ ]

8. What topics/areas of concern would you want clarified if you were to consider participating in a gene therapy clinical trial?

[ ]
