# Supplementary material for: Knowledge, attitudes, and perspectives on gene therapy for Fragile X syndrome: results from two community and caregiver surveys
Source: Front Mol Neurosci. 2026 Jul 29;19:1912516. doi: 10.3389/fnmol.2026.1912516 (PMC13461620; doi:10.3389/fnmol.2026.1912516)
Supplement: Supplementary file 3 [file Table_1.docx]

**Supplemental Table 1.** Weighted priority scores for gene therapy concerns and motivators across all top-3 selections, Survey Two (FXS caregivers, n = 56).

**(A) Gene therapy concerns**

| **Concern (Survey Two, n = 56)** | **Rank-1 n** | **Rank-2 n** | **Rank-3 n** | **Weighted Score** |  |
| --- | --- | --- | --- | --- | --- |
| **Side effects / Risks** | 26 | 14 | 10 | **116** |  |
| Effectiveness | 7 | 9 | 2 | 41 |  |
| Newness / No long-term data | 6 | 6 | 6 | 36 |  |
| Cost | 7 | 3 | 5 | 32 |  |
| Life after gene therapy | 3 | 4 | 2 | 19 |  |
| Cannot re-dose in future | 2 | 2 | 1 | 11 |  |
| Availability | 1 | 3 | 1 | 10 |  |
| Time commitment / Travel | 2 | 1 | 2 | 10 |  |
| Gene therapy composition | 1 | 1 | 1 | 6 |  |
| Ethical concerns | 0 | 1 | 1 | 3 |  |
| Lack of diversity in clinical trials | 0 | 1 | 0 | 2 |  |
| *Weighted score = (n rank-1 × 3) + (n rank-2 × 2) + (n rank-3 × 1). Respondents selected top 3 concerns; items not ranked received 0 points.* | | | | | |

**(B) Gene therapy motivators**

| **Motivator (Survey Two, n = 56)** | **Rank-1 n** | **Rank-2 n** | **Rank-3 n** | **Weighted Score** |
| --- | --- | --- | --- | --- |
| **Improving overall quality of life** | 39 | 9 | 6 | **141** |
| Need for more effective FXS treatment | 8 | 12 | 11 | 59 |
| Potential to no longer need other treatments | 5 | 10 | 7 | 42 |
| Improving care for future individuals with FXS | 2 | 7 | 14 | 34 |
| Treating a particular symptom | 3 | 5 | 4 | 23 |
| *Weighted score = (n rank-1 × 3) + (n rank-2 × 2) + (n rank-3 × 1). Respondents selected top 3 motivators; items not ranked received 0 points. FXS = Fragile X syndrome; QOL = quality of life.* | | | | |

**Legend.** Weighted scores: rank-1 = 3 pts, rank-2 = 2 pts, rank-3 = 1 pt; unranked items = 0. Scores summed across all respondents. Panel A: side effects/risks led all concerns (score 116), more than twice the next-ranked concern (effectiveness: 53). Panel B: improving QOL was the leading motivator (score 138), nearly twice the next-ranked (more effective FXS treatment: 77). FXS = Fragile X syndrome; QOL = quality of life.
